# Inter-Observer and Intra-Observer Reliability of 2D Radiograph-Based Valgus Cut Angle Measurement in Preoperative Planning for Primary Total Knee Arthroplasty

**DOI:** 10.7759/cureus.12788

**Published:** 2021-01-19

**Authors:** Anto A Gopurathingal, Sachin Bhonsle

**Affiliations:** 1 Orthopedics, Fortis Healthcare, Mumbai, IND

**Keywords:** preoperative planning, valgus cut angle, total knee arthroplasty, distal femur cut, reliability

## Abstract

Background

Preoperative planning is imperative for a successful total knee replacement. Determining the valgus angle for the distal femoral cut from the preoperative 2D radiograph is a very inexpensive method that can be used to achieve the native knee alignment during a total knee arthroplasty (TKA).

Objective

The aim of this study was to document the intra-observer and inter-observer reliability of the valgus angle determination for distal femur cut from a preoperative digital radiograph in a TKA.

Methods

A total of 20 patients with bilateral grade 3-4 primary osteoarthritis were assessed independently by a medical student, five surgeons, of which one was a consultant with more than 20 years of experience, and four residents in varied levels of training. Full-length (pelvis to toes) weight-bearing radiographs of both lower limbs were obtained prior to the surgery. The measurements were made thrice at more than 24 hours interval without any possible knowledge of their own previous measurement or that of other surgeons. We assessed the angle between the mechanical and anatomical axis of both femurs.

Results

The single measures intraclass correlation was found to be 0.733, showing that there is a moderate reliability among the six raters when single measures were considered, whereas the average measures intraclass correlation was found to be 0.943, showing that there is an excellent reliability among the six raters when average measures were considered.

For intra-observer reliability, the single measures intraclass correlation showed that there is a good-to-excellent reliability between the three trials when single measures was considered. The averages measures intraclass correlation showed that there is an excellent reliability between the three trials when average measures was considered.

Conclusion

A very inexpensive method of determining valgus angle for distal femur cut was found to have a moderate-to-excellent inter-observer reliability and a good-to-excellent intra-observer reliability.

## Introduction

Restoration of knee alignment is a key factor in determining the outcome of total knee arthroplasty [1]. Maintaining proper alignment in the coronal plane, which is essentially restoration of the mechanical axis (MA), which decreases polyethylene wear and loosening of the component, is by far the most common reason for a failed total knee replacement [2]. Intra-operative alignment is achieved with bone resections and appropriate soft tissue releases. On the femoral side, a distal femoral cut made perpendicular to the MA restores the axis of the limb.

The commonly used methods to resect the femur perpendicular to its MA depend on the surgeon. Most surgeons routinely use a fixed valgus cut angle of 5° or 6° or may determine the valgus resection angle of each individual patient from a preoperative full length standing long-leg radiograph [3], or navigation maybe used [4]. Due to variation in individual knee anatomy, a fixed valgus resection angle may not produce an MA that passes through the center of the knee in a significant number of patients [5]. Measuring the valgus cut angle is a relatively inexpensive affair.

The valgus cut angle is same as the distal femoral valgus angle (FVA), which is the angle between the anatomical and mechanical axes of the femur. FVA is measured on a standing full length (pelvis to toes) on plain radiograph. The valgus cut angle is set onto the distal femoral resection jig to get a distal femoral cut perpendicular to the MA.

The purpose of this study is to document the intra-observer and inter-observer reliability of the FVA measurement from preoperative digital radiographs in patients planned for total knee replacement.

## Materials and methods

**Figure 1:**
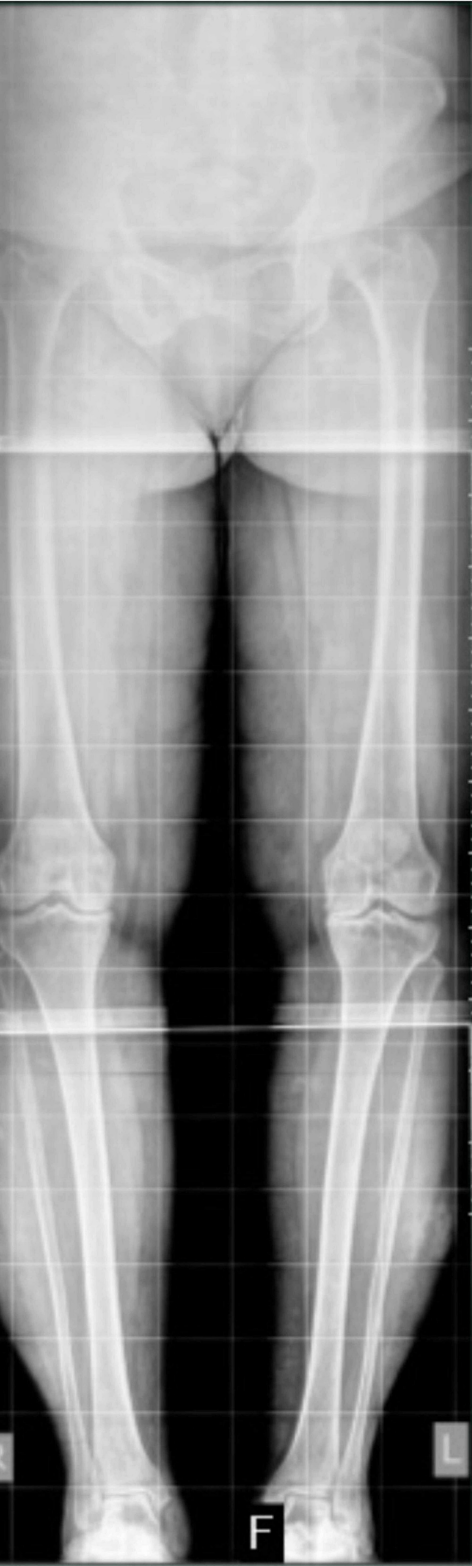
Standing scanogram

We retrospectively reviewed long leg standing radiographs of 20 patients with bilateral Kellgren-Lawrence grade III-IV primary osteoarthritis who underwent bilateral knee replacement in a single sitting. All patients were admitted under surgeons who routinely obtain long leg radiographs as part of the preoperative workup for total knee replacement (Figure 1). All radiographs were taken with two 17 x 14 inch cassettes behind the patient and stitched using the AGFA\CR software (AGFA NV, Mortsel, Belgium), with machine setting in 75 kV 30 mA owing to the distance of the tube from the patient, with the patient in a patella forward position. Up to 40° of rotation of the limb showed to have little effect on measurement of the FVA [6].

**Figure 2:**
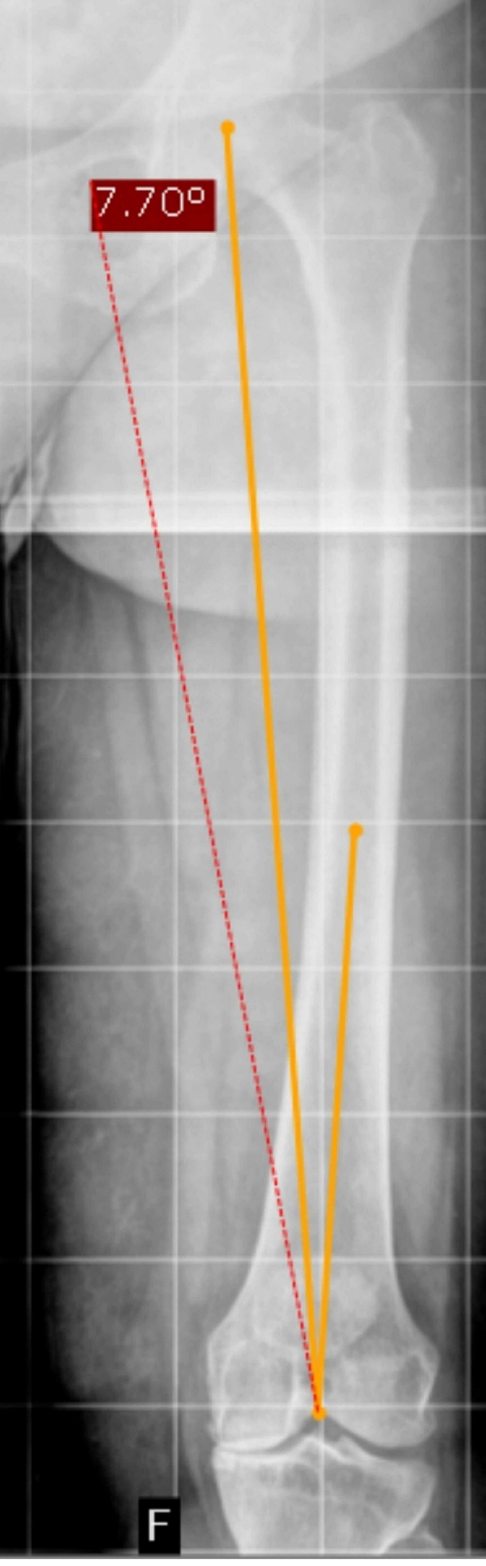
Valgus angle measured with a drawing tool in PACS PCAS, picture archiving and communication system

A total of 20 patients were randomly selected. All radiographs were included in the study. Potential difficult radiographs with deformities, rotations, and poor visibility of landmarks were included. Each radiograph was measured on a windows desktop with picture archiving and communication system (PACS) of the institution using RADspeed Viewer Version 3.5.7 (Shimadzu Corporation, Kyoto, Japan). Locating the center of the femoral head was left to individual expertise; the center of the knee was defined as the highest point of the intercondylar notch of the femur [7]. The MA of the femur was drawn as a line between the center of the femoral head and the center of the knee. The anatomical axis of the femur was drawn from the center of the knee along the center of the medullary canal as far as it would go as a straight line, mimicking the intramedullary guide during the procedure. The angles subtended by each of the anatomical and mechanical axes were measured (Figure 2). These represent the valgus resection angle that the cutting jig for the distal femoral cut would be set at during surgery.

Measurements were taken independently by one medical student, five surgeons, of which one was a consultant with more than 20 years of experience, and four residents in different levels of training. The measurements were made thrice at more than 24 hours interval between each measurement by each individual for all 20 patients without any possible knowledge of their own previous measurement or that of other surgeons. Intraclass correlation (ICC) was carried out for the values obtained.

ICC values less than 0.5 indicate poor reliability, values between 0.5 and 0.75 indicate moderate reliability, values between 0.75 and 0.9 indicate good reliability, and values greater than 0.90 indicate excellent reliability (Table 1) [8].

**Table 1:** ICC value and interpretation [8] ICC, intraclass correlation

ICC value	Interpretation
Less than 0.5	Poor reliability
0.5 to 0.75	Moderate reliability
0.75 to 0.90	Good reliability
Greater than 0.90	Excellent reliability

## Results

Inter-observer variability

**Figure 3:**
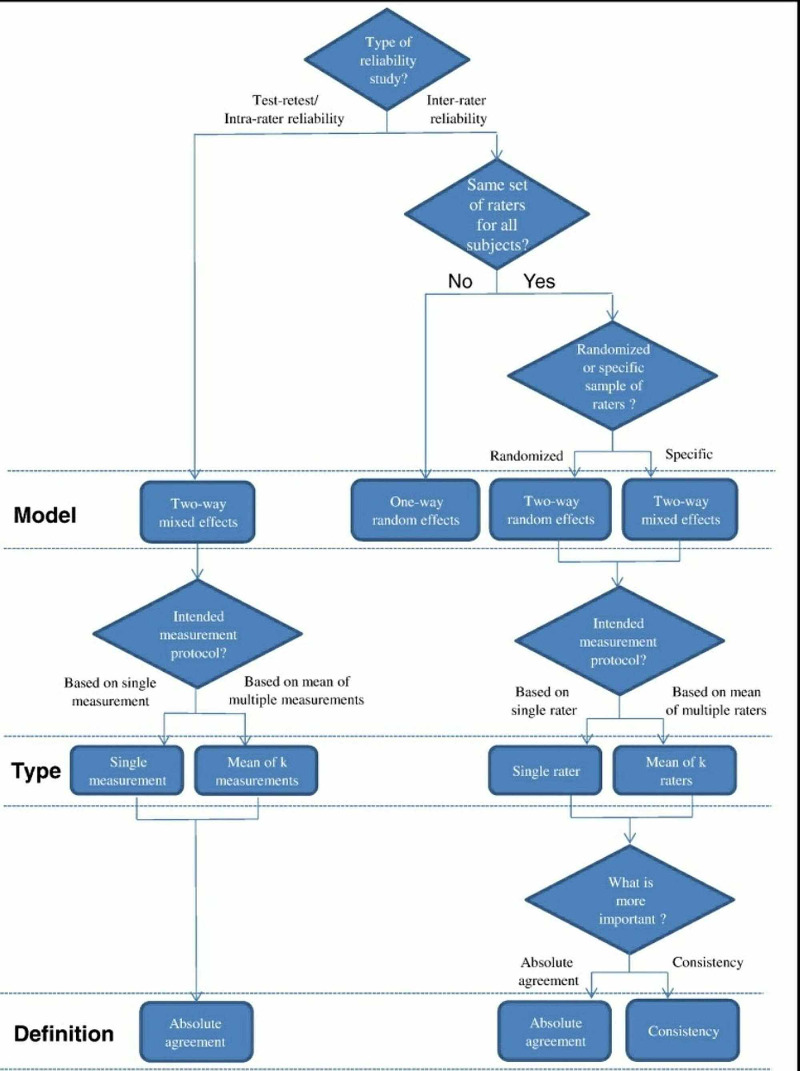
Flow chart [8]

Two-way mixed effects model was applied, where people effects were random and measures effects were fixed (Figure 3). Type A ICC coefficients were calculated using an absolute agreement definition.

The single measures ICC was found to be 0.733, showing that there is a moderate reliability among the six raters when single measures were considered. The average measures ICC was found to be 0.943, showing that there is an excellent reliability among the six raters when average measures were considered (Table 2).

**Table 2:** Absolute agreement between the six raters using intraclass correlation

Parameter	Intraclass Correlation	Interpretation
Single measures	0.733	Moderate reliability
Average measures	0.943	Excellent reliability

We calculated inter-observer reliability between the senior consultant and the medical student. Two-way mixed effects model was applied, where people effects were random and measures effects were fixed. Type A ICC coefficients were calculated using an absolute agreement definition.

The single measure ICC was found to be 0.919, showing that there is an excellent reliability between the medical student and consultant when single measures was considered. The average measures ICC was found to be 0.958, showing that there is an excellent reliability between the medical student and consultant when average measures was considered (Table 3).

**Table 3:** Absolute agreement between the medical student and the consultant using intraclass correlation

Parameter	Intraclass Correlation	Interpretation
Single measures	0.919	Excellent reliability
Average measures	0.958	Excellent reliability

Intra-observer variability

Two-way mixed effects model was applied, where people effects were random and measures effects were fixed. Type A ICC coefficients were calculated using an absolute agreement definition.

Assessor 1

In the readings of the first assessor, the single measures ICC was found to be 0.925, showing that there is an excellent reliability between the three trials when single measures was considered. The averages measures ICC was found to be 0.974, showing that there is an excellent reliability between the three trials when average measures was considered (Table 4).

**Table 4:** Absolute agreement between three trials conducted by the medical student using intraclass correlation

Parameter	Intraclass Correlation	Interpretation
Single measures	0.925	Excellent reliability
Average measures	0.974	Excellent reliability

Assessor 2

In the readings of the senior consultant, the single measures ICC was found to be 0.945, showing that there is an excellent reliability between the three trials when single measures was considered. The averages measures ICC was found to be 0.981, showing that there is an excellent reliability between the three trials when average measures was considered (Table 5).

**Table 5:** Absolute agreement between three trials conducted by the consultant using intraclass correlation

Parameter	Intraclass Correlation	Interpretation
Single measures	0.945	Excellent reliability
Average measures	0.981	Excellent reliability

Assessor 3

In the readings of junior resident 1, the single measures ICC was found to be 0.953, showing that there is an excellent reliability between the three trials when single measures was considered. The averages measures ICC was found to be 0.984, showing that there is an excellent reliability between the three trials when average measures was considered (Table 6).

**Table 6:** Absolute agreement between three trials conducted by junior resident 1 using intraclass correlation

Parameter	Intraclass Correlation	Interpretation
Single measures	0.953	Excellent reliability
Average measures	0.984	Excellent reliability

Assessor 4

In the readings of junior resident 2, the single measures ICC was found to be 0.925, showing that there is an excellent reliability between the three trials when single measures was considered. The averages measures ICC was found to be 0.974, showing that there is an excellent reliability between the three trials when average measures was considered (Table 7).

**Table 7:** Absolute agreement between three trials conducted by junior resident 2 using intraclass correlation

Parameter	Intraclass Correlation	Interpretation
Single measures	0.925	Excellent reliability
Average measures	0.974	Excellent reliability

Assessor 5

In readings of senior resident 1, the single measures ICC was found to be 0.996, showing that there is an excellent reliability between the three trials when single measures was considered. The averages measures ICC was found to be 0.988, showing that there is an excellent reliability between the three trials when average measures was considered (Table 8).

**Table 8:** Absolute agreement between three trials conducted by senior resident 1 using intraclass correlation

Parameter	Intraclass Correlation	Interpretation
Single measures	0.996	Excellent reliability
Average measures	0.988	Excellent reliability

Assessor 6

In the readings of senior resident 2, the single measures ICC was found to be 0.886, showing that there is a good reliability between the three trials when single measures was considered. The averages measures ICC was found to be 0.959, showing that there is an excellent reliability between the three trials when average measures was considered (Table 9).

**Table 9:** Absolute agreement between three trials conducted by senior resident 2 using intraclass correlation

Parameter	Intraclass Correlation	Interpretation
Single measures	0.886	Good reliability
Average measures	0.959	Excellent reliability

## Discussion

Careful preoperative planning and achieving appropriate alignment, sizing, and ligament balance are critical for optimal knee function [9]. Distal femur cut has been in much debate since the 1990s. Kharwadkar et al. [10] investigated the safety of cutting distal femur at 5°-6° in uncomplicated primary knee replacement. They calculated a mean angle of 5.4° between the anatomical and MA of the femur, with a 95% confidence interval of 5.2° to 5.6°. Even though the recommendation of the study was that it was safe to make a routine cut, we, as well as other authors, feel that it may lead to altered alignment in a substantial percentage of cases. McGrory et al. [3] addressed the question of whether preoperative radiographs in total knee replacement improved the postoperative alignment of the knee. They concluded that there was no difference in the postoperative alignment with or without the use of a preoperative long leg radiograph. Many authors have researched and found the superiority of CT planning for the distal cut compared to the 2D plain long leg radiograph [11,12].

We observed that most variability in the measurement happened in radiographs which had (a) poor visibility of the head of the femur, (b) bowed femur, (c) rotated films causing difficulty in locating the highest point in the intercondylar notch.

**Figure 4:**
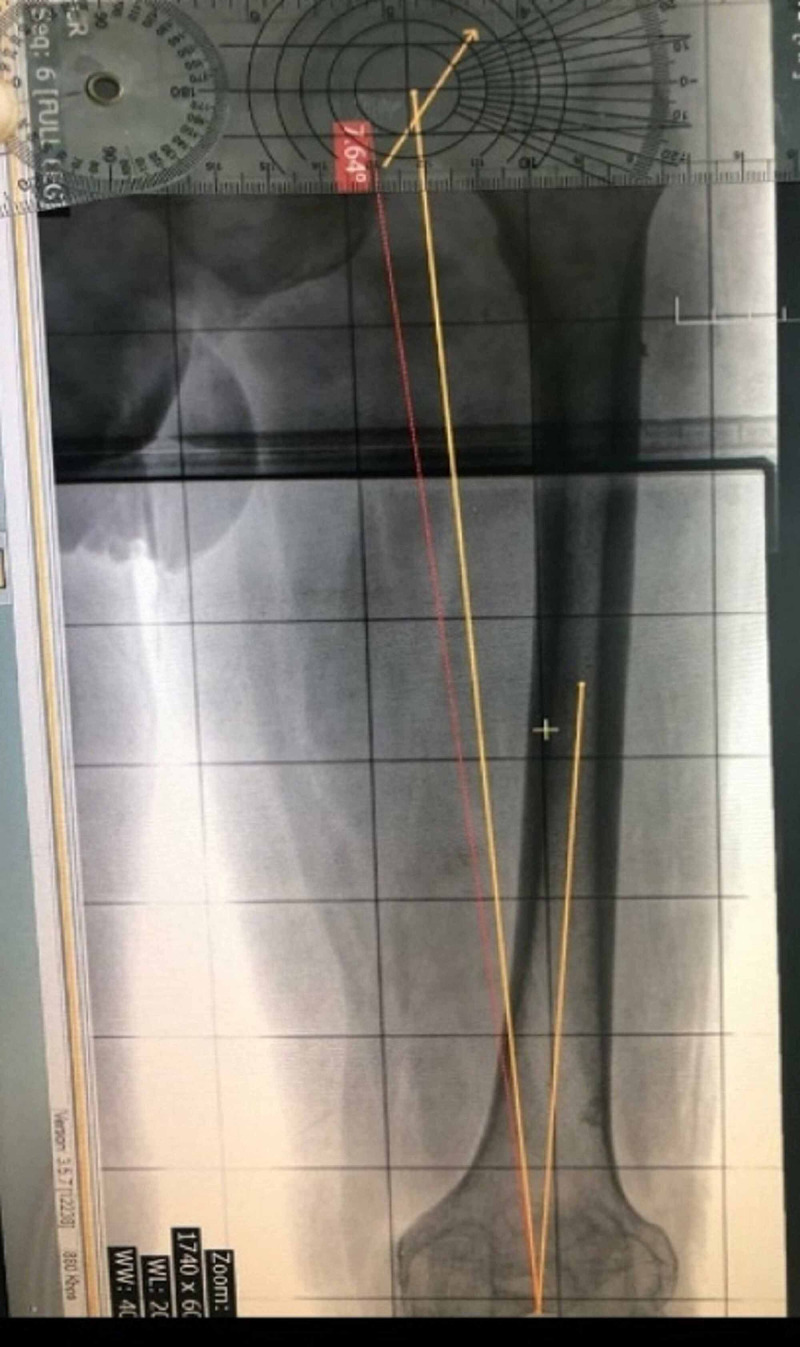
Protractor body used to find the center of the femoral head

Locating the center of the head of the femur was challenging in some of the radiographs. We feel that any geometric aid to locate the center of the head would make the absolute agreement in inter-observer and intra-observer ICC closer to 1. Cuomo et al. described Mose’s concentric circles to aid in identifying the center of the head more accurately in dysplastic hips in Perthes [12,13]. We found it easier to use the protractor body of a transparent goniometer (Figure 4) as a guide to find the center in an office setting.

Assessing the anatomical axis in a femur is always tricky because of the bowing of the shaft of the femur. Moreland [14] described anatomical axis as the connecting line between the midpoints of the medial-to-lateral width of the femoral diameter at half of femoral length and 10 cm above the joint line. Other definitions for anatomical axes have been put forward, but no significant differences were found between them [9]. Yazdi et al. [15] compared the valgus cut angle of the distal femur determined by anatomical axis of the either full length or distal half of the femur in both normal and varus aligned femurs and found a significant difference between the angles measured.

Preoperative planning on plain radiographs is very accurate if neutral rotation is guaranteed [6]. In patients with a pathologically fixed rotation secondary to osteoarthritis, deviation is possible. In such cases, it will be necessary to make an estimated correction of the measurements proportional to the degree of rotational deviation, as noted by Swanson et al. [6].

Clinical impact 

The relevance and accuracy of preoperative planning on a 3D CT is well established. Importance of such a study comes in when CT cannot be justified as a routine investigation in the preoperative workup of a total knee arthroplasty. It will add to the burden of expenditure onto an already expensive procedure. The expense in terms of time is also a factor in the modern world of overloaded operating rooms and day care arthroplasties. Largely, plain radiographs, which are relatively inexpensive and readily available, have to be depended on to calculate the valgus cut angle. Our study found a dependable inter-observer and intra-observer reliability when valgus angle was calculated on a digital platform.

Novelty of the study

With the advent of advanced imaging and diagnostic techniques, the relevance of plain radiographs is often underestimated. We feel that with proper research into how to account for the errors in calculation of parameters in plain radiographs, it can be 'use for all' tool in all centers throughout the world and a next step in standardized care in total knee replacement. We suggest using the protractor body of a transparent goniometer (Figure 4) as a guide to find the center of the femoral head in an office setting. We hope the reliability we found in the calculation of valgus cut angle would be a step towards further research in standardizing preoperative planning for total knee replacement.

## Conclusions

Preoperative planning based on a plain radiograph is an economical method of determining valgus angle for distal femur cut. It was found to have a good-to-excellent inter- and intra-observer reliability even in inexperienced hands. We followed no specific standardized protocol in lineating the mechanical and anatomical axes of the femur. We feel that with standardized methods and better tools, a better agreement can be achieved in measuring the valgus cut angle, which is a very important variable in maintaining the postoperative alignment in a total knee replacement, especially in resource-poor settings, decreasing the need of CT for a reliable preoperative planning.
